# Video education about side effects of chemotherapy and immunotherapy and its impact on the anxiety, depression, and distress level of cancer patients

**DOI:** 10.1186/s40359-022-00994-3

**Published:** 2022-11-24

**Authors:** Bohdan Baralo, Mahati Paravathaneni, Akhil Jain, Bhanusowmya Buragamadagu, Aliza Khanam, Sabah Iqbal, Samia Hossain, Sana Mulla, Eugene Choi, Rajesh Thirumaran

**Affiliations:** 1 Internal Medicine, Mercy Fitzgerald Hospital, Darby, PA 19023 USA; 2 Medical Oncology, Mercy Fitzgerald Hospital, Darby, PA USA

**Keywords:** Cancer education, Psychological distress, Anxiety, Depression

## Abstract

**Background:**

Patients diagnosed with cancer are at higher risk of anxiety, depression, and overall distress. These mood disturbances are risk factors for non-adherence to cancer treatment, increased length of stay during hospital admissions, increased number of visits to the emergency department, and also impact survival. Although paper handouts about the potential side effects are widely used in the oncology practice studies have shown that digital educational material is known to work better when compared to traditional methods. However, the impact of video education on anxiety, depression, and distress have not been previously evaluated. Our study aimed to assess whether video education about potential chemotherapy and immunotherapy can reduce anxiety, depression, and distress levels.

**Methods:**

After IRB approval, we enrolled patients who were fluent in English, younger than 80 years of age, and who were able to provide informed consent. The Hospital Anxiety and Depression Scale and Distress Thermometer were used to assess distress, as well as depression and anxiety before and after watching video educational material. Paired t-test was used to compare the differences between the scores before and after watching educational videos. The statistical software GraphPad Prism 9, San Diego, California, was used to perform the statistical analysis.

**Results:**

We enrolled 29 patients, of whom 20 completed the study, six withdrew, two were lost to follow-up, and one did not complete the initial questionnaire. Of all patients that completed the study 85% of the patient found videos helpful, and they were 7/10 likely to recommend them to other patients who may experience symptoms. The mean depression score changed from 4.75 before to 4.9 after watching the videos (p 0.77), distress score from 2.3 to 2.65 (p 0.52), and anxiety scores changed from 4.85 to 6.15 (p 0.03). The feedback provided by the patients indicated that they were more willing to watch the videos related to the side effects they experienced at their free time and convenience.

**Conclusions:**

Our study suggests that patients were open to video education and found it helpful and worth watching. However, the exposure of the patients to the videos about potential side effects of cancer treatment, including those patients do not experience, may lead to increased anxiety.

## Introduction

A diagnosis of cancer and its treatment course places additional stress on a patient, who is generally at a higher risk for increased anxiety and depression, and other mood disturbances [1, 2]. Overall, surveys have found that 20% to 52% of patients show a significant level of distress [3–5]. Distress is a risk factor for non-adherence to cancer treatment, increased hospital stay, increased frequency of visits to the emergency room, increased healthcare costs, decreased decision-making capacity, poor quality of life, and overall worsening survival. [6–15] National Comprehensive Cancer Network recognizes the importance of addressing mood disturbances and recommends including distress management programs/services in institutional continuous quality improvement projects [16]. Scientists and clinicians thus developed different approaches to managing the patient's mood disturbances. In recent years multiple promising results in reducing mood disturbances in patients were achieved by Yoga therapy [17], meditation [18], home base exercise and walking programs [19, 20], psychological interventions[21, 22], utilization of application and web services for patient-provider communication[23, 24], web-based self-management tools[25–27], and multiple educational programs. Almost all of the studies conducted regarding the education of patients with cancer have shown positive outcomes [28]. Educational programs could be delivered in paper and in person [29–32], as well as in the video format. [33–36] However, the mode of information delivery remains an essential factor. Digital education for cancer patients has been shown to decrease the anxiety level better compared to traditional educational techniques. [35]

Patient education about the nature of the disease, planned treatment, possible outcomes, and potential side effects is an essential part of every visit to the oncology provider. The patients of Mercy Fitzgerald Hospital received paper handouts explaining the most common side effects, instructions on how to recognize side effects, self care, and instructions on when to seek urgent care. The goal of this study was to assess whether the video based patient education can help reducing the level of distress, anxiety and depression in patient who were currently receiving active cancer treatment. The video course contained short educational videos that elaborated on the potential side effects of chemotherapy and immunotherapy.

## Methods

Seventeen videos were recorded for the current study. Each video ranged between 2 to 4 min long and devoted to a specific side effect. The following topics were elaborated: anxiety, constipation, depression, dehydration, diarrhea, insomnia, taste changes, eye changes, fatigue, hair loss, nail changes, nausea, mouth sores, skin changes, thrombocytopenia, leukopenia, a respiratory infection. The video had information about the nature of the potential side effect, its recognition, non-medical management, and further instructions when to seek urgent care from medical providers. In the current study, we included patients who were 18 years or over who were willing to participate and capable of providing informed consent while undergoing active treatment within the Mercy Fitzgerald Hospital infusion center. Prisoners, employees and patients older than 80 years old, patients with cognitive impairment were excluded. The study was performed in accordance with the principles of the Declaration of Helsinki and the study design was approved by the Trinity Health Mid-Atlantic Institutional Review Board on Nov, 12 2020. Informed consent was obtained from all the patients prior to enrollment. The patients who provided consent and were included in the study were asked to fill out the Hospital Anxiety and Depression Scale (HADS-T) [37]and distress thermometer (DT) [16] questionnaires. HADS contains 14 questions used to describe distress level and anxiety (HADS-A;7 questions) and depression (HADS-D;7 questions) state. This is a validated screening tool, which accuracy was also assessed in cancer patients [38–40]. The HADS-T score is considered abnormal for patients who scored 11 and above [37, 40]. There are no single accepted cutoffs for HADS-A and HADS-D subcomponents of the test [39, 40]. However, the results of a more recent study reported by Annunziata et al. showed that a score above 9 might serve as a positive result for HADS-A and scores above 7 for HADS-D subcomponents [41]. DT is an 11-point Linkert scale that ranges from 0 (no distress) to 10 (extreme distress). It resembles a visual analog scale for pain and is imaged as a thermometer. [16] The DT is a known tool incorporated in clinical practice and recommended by National Comprehensive Cancer Network (NCCN). [42, 43] Per the guidelines, score 4 and above is significant and requires intervention. Also, some researchers advocated using a score of 3 and above as a positive result [16, 44]. In our study, we used the cutoff per current NCCN guidelines to avoid unnecessary false-positive results. Per NCCN guidelines additional 40 questions are required for patients who scores positively. These questions help to identify whether practical, family, emotional, spiritual-religious, or physical areas of their routine life are causing distress. However, in our study, additional 40 questions were not collected in order to improve patient compliance via reduced time spent for completion. Patients used a tablet to fill out the questionnaire in the Microsoft Forms application.

After the patients completed the initial questionnaire, they were offered to watch videos during three consecutive visits to the infusion center. All patients had an access to 17 videos devoted to the most common complications of chemotherapy and immunotherapy. Patients were allowed to watch as many videos as they wished over the course of the study. However, we limited each visit to no more than 6 videos. We gave patients an autonomy to select videos based on their interest. The patient's willingness to continue participation was assessed during every visit to the infusion center. After completion of the third visit, patients were asked to fill out the HADS-T and DT questionnaire.

At the beginning of the study, every patient had a unique number to de-identify responses before and after completion. The number was used later to allow statistical analysis. Upon the completion of the study, every patient was asked anonymously to answer questions regarding the content. Patients were asked whether they found the content of the videos helpful and to rate from 1 to 10 likelihood that they will recommend these videos to other patients that experience side effects of chemotherapy or immunotherapy. Also, we asked every patient to report the number of videos they watched during participation in the study.

This study has pre-post test design. The comparison of scores was performed using paired t-tests. Given the study design we determined the sample size comparing paired proportions. Given the data of previous studies suggesting improvement of the anxiety in patients undergoing the education we set the study with two-tailed analysis, alfa of 5% and desired power of 60%, proportions of shifting positive to negative 0.3 which resulted minimum number of participants to be 27. However, to account for patients uninterested to participate or to continue to participate in the study as well lost to follow-up the IRB application was filed to the IRB to include 50 participants[45].

**Fig. 1:**
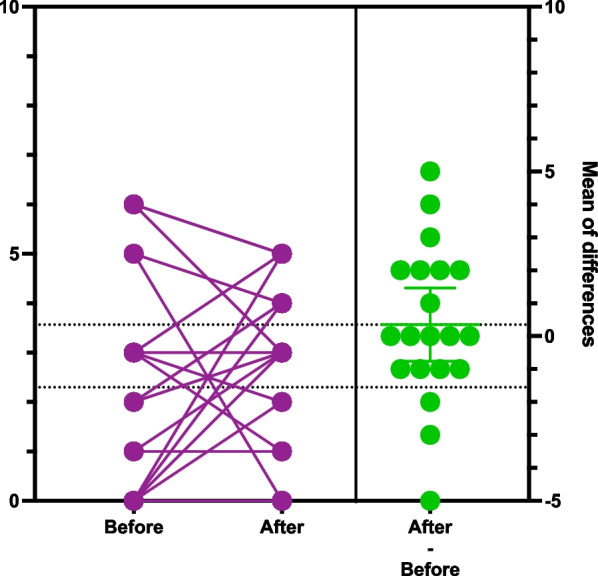
Estimation plot of Distress Thermometer scores before and after intervention

**Fig. 2:**
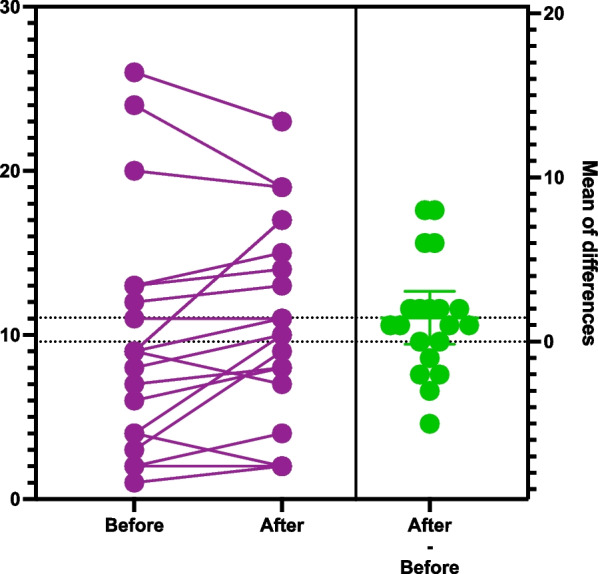
Estimation plot of total Hospital Anxiety and Depression Scale score scores before and after intervention

**Fig. 3:**
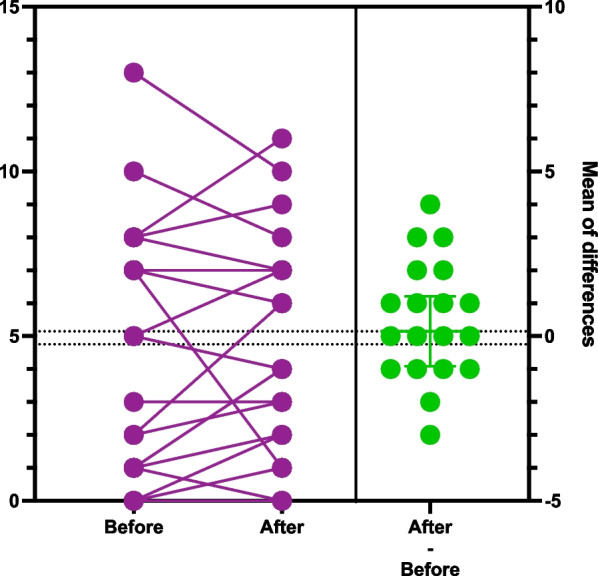
Estimation plot of depression part of HADS scale before and after intervention

**Fig. 4:**
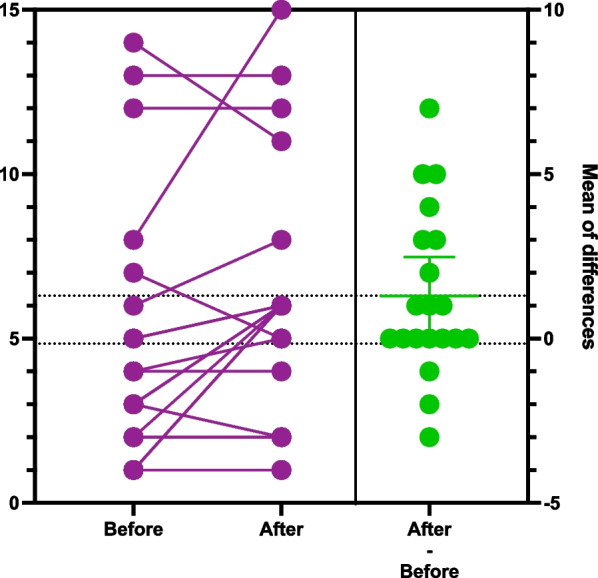
Estimation plot of anxiety part of HADS scale before and after intervention

To display the data in the graphs we chose to use the estimation plots. The score on the left y-axis represents the patient’s score on the respective tests (DT, HADS-T, HADS-A, HADS-D). All before and after intervention scores are plotted on the graph. The left Y axis represent anxiety, depression or distress scores. The lines connecting before and after score results represent every patient in the study. Benefit of current presentation allows visually analyze direction of score changes and detect individuals with significant score change. The right y-axis represent mean difference of score before and after watching videos plotted along with 95% confidence interval. Crossing of zero by the confidence interval would suggest absence of statistical significant difference (Figs. 1, 2, 3, 4) [46].

The statistical software GraphPad Prism 9, San Diego, California, was used in the current study to perform the statistical analysis [47].

## Results

After screening 50 patients as specified by the protocol, 29 patients were enrolled in the current study. Out of 29 patients, 20 patients completed the study. 6 patients withdrew from the study, 2 patients were lost to follow-up, and one did not fill out initial questionnaires. The patients enrolled in the study were African-Americans, median age of 63 years, with various location of primary solid malignancies, multiple myeloma and Hodgkin lymphoma. Majority of the patients had stage 3 and 4, with adenocarcinoma being prevalent pathology and most patient started therapy prior to enrollment in the study (Table 1). Out of 19 patients who completed the anonymous survey about the study, 84.2% found videos helpful. 3 patients watched from 0–3 videos (15.8%), 6 patients watched 7 to 9 videos (31.6%), 7 patients watched 10 to 13 (36.8%) and 3 patients 14 to 17 videos (15.8%). On average, every patient watched about 10 videos. 8 (28.5%) out of 28 patients who initially filled out the questionnaire patients had a significant elevation of DT score (≥ 4). HADS total on admission among all patients revealed 10 participants having positive screen (35.7%). 7 (25%) patients had abnormal HADS-D, and 6 patients (21.4%) HADS-A. 20 (66,67%) patients completed the study, as was specified per protocol. 10 out of 20 (50%) patients who completed the study had clinically significant HADS-T score. 4 (20%) patients had screened positive on HADS-D, and 4 patients screened positive on HADS-A score.

**Table 1:** Demographic and clinical characteristics of the patients enrolled in the study

Age (mean, SD)		62.45 ± 11.2
Sex	Male	12 (41.4%)
	Female	17 (58.6%)
Race	Caucasian	9 (31%)
	African American	19 (65.5%)
	Asian	1 (3.5%)
Location	Lung	8 (26.5%)
	Colon	4 (14%)
	Breast	4 (14%)
	Endometrial	2 (7%)
	Stomach	2 (7%)
	MM	2 (7%)
	Pancreas	2 (7%)
	Esophageal	1 (3.5%)
	Prostate	1 (3.5%)
	Cervix	1 (3.5%)
	Hodgkin	1 (3.5%)
	Bladder	1 (3.5%)
Pathology (solid)	Adenocarcinoma	24 (82.8%)
	Small cell carcinoma	1 (3.5%)
	Squamous cell carcinoma	1 (3.5%)
Stage (for solid)	Stage 2	1 (3.5%)
	Stage 3	10 (34.5%)
	Stage 4	12 (41.8%)
Previously treated	Yes	21 (27.6%)
	No	8 (82.4%)
Treatment type	Chemotherapy alone	23 (79.3%)
	Chemotherapy plus immunotherapy	6 (20.7%)

Initially, it may seem that the DT score increased after watching the videos. However, comparing the scores of 20 patients who completed the study shows that 5 out of 20 patients had clinically significant scores (25%). Through the study, 6 patients had their scores decreased, 5 patients did not change the scores, and 9 patients had their scores increased (Fig. 1. Estimation plot of Distress Thermometer scores before and after the intervention). The mean DT score before the study was 2.3 compared to 2.65 after completion, thus no statistical significance was detected (p 0.52).

The assessment of the initial HADS-T among patients who completed the study showed that seven patients screened positive, representing 35%. As we already mentioned, upon the completion, 10 patients (50%) screened positive. During the study period, 13 patients had their score increased, 5 patients had their scores decreased, and 2 patients had no score changed. The median score before completion of the study was 9.6 and 11.0 after completion. Although we noticed an increase, it was not statistically significant (p 0.08). (Fig. 2. Estimation plot of total Hospital Anxiety and Depressions Scale scores before and after the intervention).

When factoring in the analysis only patients who completed the study, the number of patients with positive HADS-D score was 5 (25%) before intervention. The number of patients with positive HADS-D results decreased after the intervention, with a total of four (20%). Through the study, 7 patients had their scores decreased, 4 patients did not have a change in the scores, and 9 patients had their scores increased (Fig. 3. Estimation plot of depression part of Hospital Anxiety and Depression Scale scores before and after the intervention). The mean depression score increased from 4.75 prior to intervention to 4.9 post-intervention (p 0.77), though showing the absence of statistically significant change.

When performing paired analysis for the patient who completed the study number of patients with clinically significant anxiety was 3 (15%) before intervention. The number of patients with clinically significant HADS-A scores increased from 3 to 4 (20%) at the end of the study. Through the study, 3 patients had their scores decreased, 7 patients did not have a change in the scores, and 10 patients had their scores increased (Fig. 4. Estimation plot of anxiety part of Hospital Anxiety and Depression Scale scores before and after the intervention). The mean anxiety score before the intervention was 4.85 and post-intervention 6.15 (p 0.03), showing a statistically significant increase in anxiety scores. Upon close review of the estimation plot and tables for paired scores prior to and after the intervention, it was noted that most often, the score was within one to five points, except for the one participant whose score increased by 7 points. When the analysis was recalculated, excluding this patient, the mean score prior to the intervention was 4.68 and 5.68 after the intervention (p 0.06), which does not reach statistical significance.

In the current study, we enrolled fewer patients than expected. However, given the magnitude of the change and absence of the desired direction in score change the increase number of participants to 27 would not help to achieve our primary end point or change overall interpretation of the study.

## Discussion

Among the 28 patients who were enrolled in the study and completed the initial questionnaire, 20 patients (71.4%) completed the full study protocol (i.e., viewed videos, completed post-video questionnaires). Only 6 patients withdrew from the study due to a lack of interest (21.4%). The last 2 patients were lost to follow-up. Most of the patients who completed the study found the videos helpful (84.2%). They gave a 7/10 likelihood that they would recommend them to other patients who had experienced the symptoms covered within the video series offered during this study.

We did not require or set up the minimum requirement for videos to be watched, and the patients were allowed to select videos they were interested in. At the end of the study, 16 patients (out of 19 who completed an anonymous questionnaire) reported that they watched more than seven videos, which represents a good interest in the topics covered within the video education course.

The distress level assessed at the beginning of the study showed that eight (28.5%) patients met the criteria for a significant distress level. Similar results were reported by Jewett et al., who assessed distress in women with gynecologic cancer ad found that 30% of patients scored 4 or above [48]. In our study among the patients who completed the protocol, the number of patients with significant DT increased from five to seven. The mean score change was 0.35, indicating a nonsignificant change. This was likely related to the fact that many patients had a score change throughout the study (nine patients had their scores increased, while six had their scores declining).

At the time of enrollment, 10 patients (35% of the study population) had positive HADS-T scores. The HADS-T initially considered 11 points as the cut-off for clinically significant distress. The result of a study within one of Missouri's safety net hospitals showed that up to 55% of patients might have scores above 10 on initial presentation [49]. However, multiple recent studies have reported higher HADS-T cut-off values. For example, Civilotti et al. in 2020 used scores above 14 as clinically significant, and 45% of the patients were positive even with a higher threshold [4, 50].

A significantly elevated HADS-D score was detected in 7 patients (25%). These data correspond to the results of the meta-analysis by Michell et al., which showed that the prevalence of any type of depression ranged from 20.7% (12.9 to 29.8%). [51]. The study results of 10,153 patients reported by Linden et al. showed that 12.9% of a patient were diagnosed with depression, and the rate ranged from 8.4% in patients with skin cancer to 17.9% in patients with lung cancers[1]. When patients who completed the study were reviewed, the number of patients with positive HADS-D screening results decreased from five to four. However, mean scores when compared showed that mean depression scores were minimally higher after the intervention (delta 0.15), thus showing no statistical significance.

The review of the HADS-A scores reported by patients who were enrolled and completed the initial questionnaire showed a positive screen in six patients (21.4%). The number of patients with anxiety states was higher than that in the meta-analysis reported by Mitchell et al., 9.8% (6.8–13.2%) [51]. However, the large-scale study reported by Lindel et al. and discussed above showed that 19.0% of patients screened positive for anxiety, and the rate ranged from 12.4% in patients with skin cancer to 28.4% in patients with gynecological malignancies [1]. When reviewing the population that completed the study, we observed that the number of patients with positive HADS-A scores increased from three to four patients). The difference between mean scores was also higher after the intervention with delta 0,3 and p 0.03, which showed a significant increase in anxiety scores, even though the mean score after intervention remained lower than the cutoff for the borderline score (8). While reviewing the estimation plot for anxiety, we also noted that the mean score increased significantly because one patient's score increased from 8 to 15. While recalculating the difference between means, excluding this patient, the delta changed to 1,0 with p 0.06, thus showing no statistically significant changes. This effect would likely be reached if the number of participants were further increased in our study.

As the study progressed, the patient provided ongoing feedback. Thus, many patients reported that they would be interested in watching videos of the side effects they are experiencing. Another significant request from patients was the ability of the patients to have access to the videos at their time and place of convenience.

The main limitation of the current study is pre-post test design. Current design leads to several possible biases. Among them are history bias (the longest time between the pretest and the post-test, the higher risk is for factors other than intervention to bias the results), maturation (for example worsening or improvement of the disease can bias study results), testing bias (the familiarity of subject with study tools makes inadvertent changes on the repeated measurement), instrumentation (fatigues, loss of interest, improvement of investigator skills introduce an opportunity for biases), loss to follow up, regression to the mean (in case of unusually high or low scoring on initial test the result tend to regress to the mean on subsequent testing) [52].

## Conclusion

The data obtained at the beginning of the study regarding the level of distress measured by DT and HADS-T among the patient population of the infusion center of our hospital corresponded to the data reported by the researchers. The number of patients who screened positive on the HADS-D and HADS-A subtests corresponds to the prevalence of depression and anxiety reported by meta-analyses and extensive studies including over 10,000 patients. Even though the study did not reach the planned number of participants, a further increase will not lead to the desired outcome, so further enrollment was not requested.

Most of the patients who participated in our study found the video education about the potential side effects of chemotherapy and immunotherapy helpful. In addition, there is a high likelihood of patients recommending videos to others experiencing the side effects covered in these video modules. There was no significant change in the depression and distress scores as a result of the intervention. The HADS-A score was higher in patients who watched the videos. This may be due to the videos exposing patients to additional side effects that they may not have personally experienced. The results of our study concluded that video education about the side effects of cancer treatment may lead to an increase in anxiety. One consideration is that patients access educational videos at the time and place of their convenience, when they can focus their attention on the material being delivered. The other is to emphasize the side effects experienced by the patients. We propose that this new approach and its impact on anxiety, depression, and distress levels should be evaluated in future large randomized studies.

## Data Availability

All data generated or analyzed during this study are included in this published article.
